# Induced Pluripotent Stem Cell Technology and Direct Conversion: New Possibilities to Study and Treat Parkinson’s Disease

**DOI:** 10.1007/s12015-012-9369-4

**Published:** 2012-04-13

**Authors:** Reinhard Roessler, Erik Boddeke, Sjef Copray

**Affiliations:** Department of Neuroscience, Medical Physiology, University Medical Center Groningen, University of Groningen, A.Deusinglaan 1, 9713 AV Groningen, The Netherlands

**Keywords:** Induced pluripotent stem cells, Parkinson’s disease, Dopaminergic neurons, In vitro disease modeling, Cell based therapy, Transdifferentiation

## Abstract

Recent developments in in vitro disease modeling and regenerative medicine have placed induced pluripotent stem cells (iPSCs) in the center of attention as a unique source to study Parkinson’s disease. After only 5 years of intensive research, human iPSCs can be generated without viral integration and under xeno-free conditions. This, combined with increasingly sophisticated methods to differentiate iPSCs into functional dopaminergic (DA) neurons, led us to recapitulate the most important findings concerning the use of iPSC technology as a prospective tool to treat symptoms of Parkinson’s disease as well as to obtain insight in disease related cell pathogenesis. Moreover, we touch upon some of the latest discoveries in which patient-derived autologous DA neurons come into even more direct reach thanks to a method that allows transdifferentiation of fibroblasts into DA neurons.

## The Rise of Induced Pluripotency

The discovery of induced pluripotent stem cells quickly developed into one of the most competitive and most sophisticated research areas in biology. In 2006, a key study by the group of Shinya Yamanaka showed for the first time that somatic cells, such as murine embryonic fibroblasts (MEFs), could be reprogrammed to a pluripotent, embryonic stem (ES) cell-like state [1]. This epigenetic reprogramming event in fibroblasts was driven by viral delivery of four transcription factors (hereafter referred to as ‘Yamanaka factors’), namely Oct4, Sox2, Klf4 and cMyc. It became clear that when these genes successfully integrated into the genome of a host cell and expression occurred in an optimal stochastic manner, the virus transfected cell changes from a differentiated somatic cell into a pluripotent stem cell, which in turn is able to differentiate into every cell type of the body. Shortly after this groundbreaking discovery several other groups confirmed and reported the generation of iPSCs. Procedural optimization steps have been undertaken thereafter for example by using a more suitable selection marker for the reprogrammed induced pluripotent stem cells Oct4 or Nanog [2] instead of Fbx15 (used by the Yamanaka group) or even by identifying iPSC colonies merely on their morphology instead of the use of genetically modified fibroblasts as starting material [3]. IPSCs generated by this epigenetic reprogramming process have been characterized and identified as truly fulfilling the criteria for pluripotency within a very short time span. Thus, iPSCs were not only able to differentiate into cell types from all three germ layers (mesoderm, endoderm and ectoderm) and to contribute to embryo formation after injection in a blastocyst (chimeric mice), they could also contribute to the germ line in such a setup. Moreover, they formed teratomas after subcutaneous injection or subcapsular implantation in the testis or kidney [4, 5]. Last but not least, as one of the most stringent criteria for pluripotent cells, iPSCs have been injected in a tetraploid blastocyst, which was subsequently implanted in a surrogate mother mouse, where they gave rise to so called ‘all-iPS mice’, viable mice that exclusively originated from the implanted iPSCs, via a process called tetraploid complementation [5, 6].

The next major step in iPSC research was the discovery that also human somatic cells could be reprogrammed into iPSCs using a similar approach and with similar properties as mouse iPSCs [7, 8].

Due to the extremely rapid development in this particular research field, iPSCs became highly interesting tools for in vitro disease modeling but also for potential application in regenerative medicine. However, the reprogramming procedure itself was bearing a crucial and very undesirable component. IPSCs so far were generated by use integrating viruses that intrinsically modified the genome of the host cells. Especially the integration of oncogenes such as cMyc and Klf4 would not be acceptable for any clinical application of iPSC-derived cells or tissues. In order to avoid this hurdle, several methods have been tested to circumvent viral integration events. First attempts were directed to reduce the number of integrating proviruses e.g. by eliminating Myc transduction [9], but also the generation of iPSCs with non-integrating adenoviruses and temperature sensitive Sendai viruses has been tested and proven possible [10, 11]. In a next logical follow up step, the use of viruses has been abandoned completely by utilization of direct repeated transfection with ‘Yamanaka factor’ containing plasmids [12] or piggyback transposons [13]. Another interesting approach is the use of microRNAs (miRs) to drive induction of pluripotency. Anokye-Danso et al. [14] showed that two specific miRs (miR302 and miR367) are sufficient to induce pluripotency in mouse and human somatic cells without forced expression of exogenous transcription factors [14]. Even more towards a safe clinical use is the induction of pluripotency with RNA molecules that code for the four ‘Yamanaka factors’ [15]. Eventually it has been shown that iPSCs can also be generated by direct Oct4-, Sox2-, Klf4- and cMyc- protein delivery to human fibroblasts [16].

**Fig. 1:**
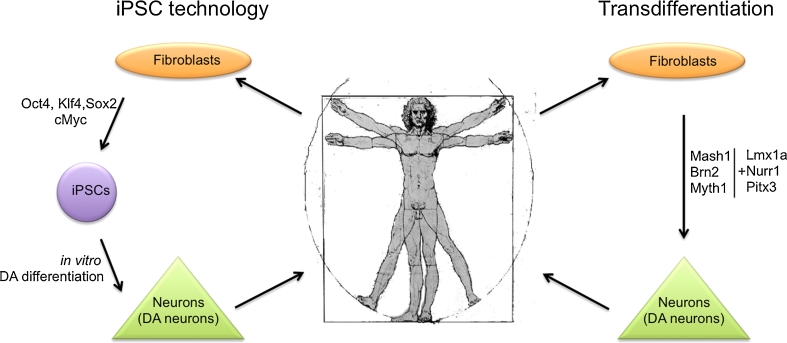
Schematic comparison between iPSC technology and trans-differentiation to generate DA neurons in vitro. IPSC technology requires a forced expression/induction (either by viral transduction, RNA or protein transfection) of Oct4, Klf4, Sox2 and cMyc. Recent DA differentiation protocols provide robust yields of neurons highly resembling DA neuron characteristics. Directly converted neurons are not derived from a pluripotent intermediate, which minimizes undesired differentiation potential and risks for teratoma formation. DA-like neurons generated so far however show only very limited resemblance with primary midbrain DA neurons

In general it seems that technical safety issues that previously hampered the use of iPSCs for a wide variety of clinical applications are about to be solved. In the scope of this review these developments are particularly interesting for the in vitro generation of iPSC derived DA neurons (Fig. 1).

## Mouse iPSCs as Crucial Model System

Mouse as well as human iPSCs have been shown to be capable to differentiate into varying clinically relevant cell types, such as cardiomyocytes [17, 18], hepatocytes [19], hematopoetic progenitors [20], oligodendrocytes [21] and specific subtypes of neurons [22, 23]. In particular, iPSC-derived DA neurons appear to be a very interesting, clinically relevant cell type (for overview of key studies see Table 1). First, they might serve as novel easily accessible autologous source for cell replacement. Many studies, starting in the 1980s, have already demonstrated that the consequences of the specific loss of midbrain DA neurons in the substantia nigra pars compacta (SNc), the hallmark of Parkinson’s disease, can be partly obviated by the intrastriatal implantation of extrinsic (heterologous) abortion-derived fetal human DA neurons [24–26]. Although this approach itself appeared to be successful, major practical and ethical concerns related to the use of this fetal human graft source were unbridgeable and made this strategy not feasible for the clinic [27, 28].

**Table 1:** Key studies for in vitro generation of DA neurons/iDA neurons from pluripotent stem cells and somatic cells (trans-differentiation), respectively

Species	Viral integration	Pluripotent stem cells	Characterization	Reference

Mouse	Yes	iPSCs	DA markers, electrophys.properties, functional integration	[22]
Human	No (direct protein delivery)	human iPSCs	DA markers, electrophys. Properties, DA release, functional integration	[51]
Human	Yes(Cre-excised)	iPSCs (patient specific)	DA markers	[52]
Human	Yes(Cre-excised)	iPSCs (patient specific)	DA markers, functional integration (6OHDA rats)	[53]
Human	No (Sendai virus)	ESCs/iPSCs	Floor plate based DA induction, DA gene expression profile, electrophys. properties, functional integration	[39]

Species	Viral integration	Trans-differentiation	Characterization	Reference

Mouse	Yes	transdiff. fibroblasts iDA neurons	DA markers (TH selection) electrophys.properties, DA release, global gene expression (TH sorted), functional integration	[62]
Mouse	Yes	transdiff. fibroblast iDA neurons	DA markers (Pitx3 selection), electrophys.properties, DA release, selected gene expression (Pitx3 sorted), functional integration	[69]

Secondly, iPSC derived DA neurons provide a unique tool to investigate cell pathogenic mechanisms in detail (e.g. the role of α-synuclein or LRRK2, etc.), particularly when the iPSCs are generated from patients with a hereditary form of Parkinson’s disease [29, 30].

Before the iPSC technology entered the playground, DA neuron differentiation has been extensively studied for embryonic stem cells (ESCs) and neural stem cells (NSCs), either derived from ESCs [31, 32] or primary isolated [33, 34]. In order to improve in vitro generation of DA neurons derived from ESCs protocols have been developed that enhance DA differentiation. Early approaches involved the use of stromal feeder layers (such as MS5 or PA6 cells) [35, 36]. Later, in order to avoid undefined factors, protocols have been established that employ small molecules and recombinant proteins which specifically inhibit BMP signaling pathway (dual inhibition of SMAD signaling) [37]. Recent studies provide evidence that derivation of floor plate cells *(the floor plate is a crucial organizing structure in the developing embryo, located along the ventral midline)* from pluripotent stem cells, by early supplementation with high concentrations of sonic hedgehog and induction of canonical Wnt signaling by small molecules, drastically improves the quantity and quality of subsequently generated DA neurons [38, 39].

Based on established methods for in vitro differentiation of DA neurons efficient protocols for dopaminergic differentiation of iPSCs have been developed. One of the first studies by Wernig et al. [22] showed successful generation of DA neurons derived from mouse iPSCs [22]. These cells showed some specific DA marker expression, such as the transcription factors Nurr1, Pitx3 and the enzyme tyrosine hydroxylase (TH). Furthermore, they revealed typical neuron-like electrophysiological properties and they functionally integrated in a rat model for PD after transplantation. However, the number of DA neurons that could be generated did not exceed 4 % of the starting iPSC population, indicating that most of the cells that developed with that differentiation procedure had non-DA characteristics. While the study provided the proof of principle for treating PD symptoms with iPSC derived DA neurons in an animal model, various important issues were not or could not be addressed. Firstly, since only a minority of grafted iPSC derived cells were DA neurons, it can be questioned what the effect was of the vast majority of other cell types in the graft; it is clear that some kind of purification step is required. Secondly, in view of the exceptional origin of the DA neurons, characterization of the iPSC derived DA neurons and assessment of the completeness and stability of differentiation should not only be based on a set of general DA markers and electrophysiological characteristics, but should also contain extensive genetic and epigenetic screening. The challenge for a more comprehensive study of epigenetic and genetic characteristics of iPSC derived DA neurons mainly lies in the necessity to generate a cell population that allows purification based on a highly specific midbrain DA marker. Earlier studies reported the generation of ESC lines with specific heterozygous GFP knock-in modification in the dopamine transporter (DAT) locus and the Pitx3 locus [40, 41]. Both genes code for specific DA markers. Pitx3 is a transcription factor specifically expressed in mDA neurons, that interacts with Nurr1 and is crucially involved in differentiation and maintenance processes for mDA neurons [42–45]. DAT is widely expressed in DA neurons and specifically indicates their maturation [46]. However, because of its general function in DA neurons (residing e.g. in the olfactory bulb or the ventral tegmental area) DAT cannot be considered as a selective marker for mDA neurons.

**Fig. 2:**
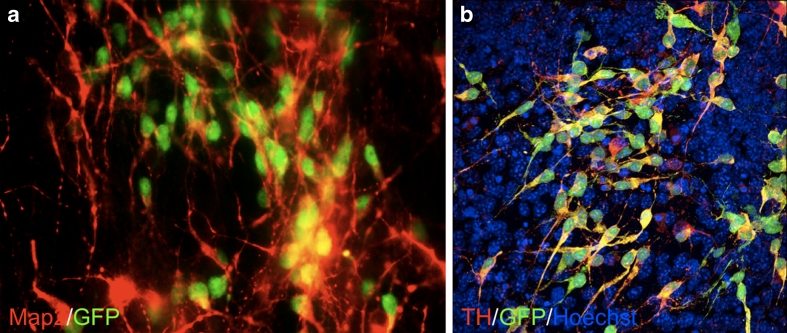
Pitx3-GFP iPSC derived mDA neurons show co-expression of transgenic GFP (Pitx3^gfp/+^) and Map2 (**a**) as well as tyrosine hydroxylase (TH) (**b**). Confocal microscopy (**b**) revealed that most of the Pitx3 expressing cells also express TH. However TH positive cells do not always show Pitx3 expression

Our group has used Pitx3-GFP knock-in mice (kindly provided by Prof. M. Smidt, SILS, University of Amsterdam) for the generation of iPSC lines. The Pitx3-GFP knock-in feature allowed us selective isolation, identification and purification of primary embryonic and postnatal mDA neurons as well as of DA neurons that were obtained via differentiation of the iPSCs (see Fig. 2). This approach enabled us to perform an extensive comparison of iPSC-derived DA neurons with primary mDA neurons (including varying developmental stages) as far as their genetic and epigenetic profiles are concerned. We particularly focused on DNA methylation, since reprogramming of somatic cells towards pluripotent cells and subsequent differentiation into DA neurons must entail massive changes in DNA methylation patterns in specific genomic loci [47–50]. This type of characterization, which was based on the ability to analyze purified Pitx3-GFP DA neurons, enabled us to determine a close similarity in terms of DNA methylation patterns between iPSC-derived DA neurons and primary DA neurons and provides novel insight in cell type specific de novo methylation during in vitro differentiation (Roessler et al., manuscript in preparation). Before human iPSC-derived DA neurons might serve as tool for future cell replacement approaches such detailed in depth-studies will certainly be required in order to assure their clinically safe status as well as their bona fide characteristics. It will however remain difficult to directly compare human iPSC-derived DA neurons tor their primary counterpart.

## Human iPSCs to Study and Treat PD

Clinical application of iPSC-derived DA neurons for treating Parkinson’s disease is still a distant option. All the aforementioned issues concerning the clinically safe use of iPSCs as well as the incomplete characterization of in vitro generated DA neurons form prominent roadblocks that remain to be cleared. A recent study provides evidence that human iPSCs completely free of proviruses (these hiPSCs were generated by direct protein delivery) are efficiently capable to differentiate towards functional DA neurons [51]. Such patient-derived iPSCs will provide a valuable tool for possible future cell based therapy approaches.

Soldner et al. [52] were the first to generate human iPSCs (hiPSCs) cells from patients with idiopathic PD [52]. Their study showed that indeed reprogramming factor-free iPSCs could be generated from PD patients using Cre-recombinase excisable viral constructs. Moreover, those patient-specific iPSCs could be differentiated in tyrosine hydroxylase (TH)-expressing neurons. In a follow-up study, PD patient iPSC-derived TH expressing neurons have been transplanted in a PD rat model (6-OHDA), where they reduced specific neurotoxin-induced asymmetric motor behavior [53]. These studies demonstrate the capability of hiPSCs to differentiate into functional neurons that improve PD symptoms associated with reduced dopamine signaling in the striatum. However, more extensive research needs to be done to further characterize patient-specific iPSC derived DA neurons in terms of the completeness and stability of their differentiated state. For that, global gene expression studies but also, as mentioned above, in-depth studies of epigenetic characteristics such as DNA methylation and histone modifications are required before clinical application will come within reach.

Regarding the use of patient-specific iPSC-derived DA neurons as in-vitro PD model, it should be considered that PD is in general a late onset disease, which affects patients after decades of latent disease progress. Therefore, it may be quite challenging to model PD in vitro. Slowly developing molecular changes such as α-synuclein aggregation in patient derived DA neurons might not be detectable in cell culture that at best can be maintained for a few month. Moreover, genetic variations intrinsic to patient-specific iPSCs could complicate disease modeling since it will be literally impossible to generate experimentally defined conditions. A very interesting approach in that respect is a technology known as ‘genome editing’, employing zinc finger nucleases (ZFNs) to site-specifically target a disease relevant gene. Soldner et al. [54] used ZFNs in patient-specific iPSCs to exclusively manipulate a point mutation site in the α-synuclein gene known to be key in rare forms of familial PD [54]. Mutations in α-synuclein at specific sites (e.g.: A53T; E46K or A30P) lead to the formation of Lewy bodies, which are inclusions present in affected DA neurons (for reviews on α-synuclein and Lewy bodies see e.g. [55, 56]). In this study by Soldner and colleagues [54] these mutations have been addressed with genome editing aiming either at correction of a specific mutation (e.g. in PD patient specific hiPSCs) or vice versa at generation of a mutation (e.g. in wild-type ES cells) in order to study cell pathogenic consequences of such disease relevant modifications.

Even though, DA neurons generated from human iPSCs do not easily allow purification as indicated for transgenic mouse iPSC-derived DA neurons, a thorough characterization is extremely crucial. Conceivable approaches also involve genome editing. ZFNs (as described above) and transcription activator-like effector nucleases (TALENs) have been used to genetically engineer human iPSCs that contain the GFP coding sequence (either 2A-eGFP or eGFP) in the locus of the Pitx3 gene [57, 58]. As yet, however no study showed the purification of Pitx3-GFP cells derived from ZFNs- or TALENs-modified human iPSCs. Succeeding in such a purification step will certainly improve the possibility to study specific molecular modifications (e.g. mutations in the α-synuclein- or LRRK2-gene) and their consequences in affected cells. It might also allow to find changes in cells bearing disease relevant modifications before phenotypic (pathological) changes are detectable.

## Towards a Direct Patient Specific Cell Replacement Strategy

Very recent studies report about the direct conversion or transdifferentiation of fibroblasts to neurons (see Fig. 1), avoiding a pluripotent ‘in between-stage’. This approach, although in a yet premature phase, shows a highly interesting option to generate DA neurons, circumventing some of the most critical pitfalls of the iPSC technology. In 2010, researchers described the possibility to manipulate mouse fibroblasts by introducing three neurodevelopmental factors (Brn2, Ascl1 and Myth1l) in such a way that they directly converted into neuronal cells, so called induced neurons, or iNs [59]. Shortly after that, the same group reported the same achievement for human fibroblasts [60]. Both, mouse and human iNs showed expression of neuronal markers such as Tuj1, Map2, Tau and synapsin and revealed neuron-like electrophysiological properties.

In a more PD relevant approach two groups showed that the combination of the above mentioned iN factors combined with Lmx1a and FoxA2 [61] or a combination of Lmx1a and Nurr1 [62] in fibroblasts is sufficient to directly induce cells with DA neuronal characteristics, therefore called iDA neurons.

The combination of the three neurodevelopmental factors (BAM) with factors known to be crucially involved in the embryonic development of mDA neurons as well as for their beneficial effect for DA differentiation of ESCs [63–68] seem to specifically boost the transdifferentiation towards DA-like neurons. Even though these findings provide a proof of principle for the amazing possibility to directly transdifferentiate/convert somatic cells just by simple forced expression of a specific set of transcription factors, also for iDA neurons a very thorough characterization would be necessary before one could seriously consider their clinical application.

For a better understanding of to what extent iDA neurons are similar to primary midbrain DA neurons, the use of transgenic Pitx3-GFP mice, as described above for iPSCs, can be an adequate tool. Kim et al. [69] used tail tip fibroblasts from transgenic Pitx3-GFP mice for transdifferentiation experiments [69]. iDA neurons were isolated based on GFP expression and gene expression was compared to primary midbrain DA neurons (equally positive for Pitx3-GFP). Comparison of a selected list of markers for DA neurons showed only limited similarities between iDA and primary DA neurons. However, Pitx3-GFP sorted iDA neurons showed dopamine release, acquired highly similar electrophysiological properties and showed functional integration in 6-OHDA lesioned mice.

Taken together, the possibility to directly convert somatic cells into neurons might provide a valuable tool to study diseases like PD in vitro. Besides, when issues such as the conversion efficiency and the not yet optimal genotypic and phenotypic characteristics are solved and improved, iDA neurons could provide a cell replacement tool as well.

## Concluding Remarks

The full scope of iPSC-based technology in terms of in vitro disease modeling as well as in regenerative medicine becomes more and more apparent. This might in particular be the case for approaches to treat Parkinson’s disease. We strongly believe that future research in this exciting field will provide much more details about disease-causing factors, at cellular as well as environmental level. In addition, a clinically safe generation of human iPSCs and the subsequent in vitro generation of patient specific DA neurons might provide realistic tools to replace lost DA neurons in PD patients. Last but not least the sheer possibility to generate specific DA neurons directly from patient fibroblasts opens up a completely new angle concerning cellular PD research.
